# Changes in children’s cardiorespiratory fitness and body mass index over the course of the COVID-19 pandemic: a 34-month longitudinal study of 331 primary school children

**DOI:** 10.1007/s12519-023-00772-0

**Published:** 2023-11-26

**Authors:** Gerald Jarnig, Reinhold Kerbl, Mireille N. M. van Poppel

**Affiliations:** 1 Institute of Human Movement Science, Sport and Health, University of Graz, Mozartgasse 14, 8010 Graz, Austria; 2 Department of Pediatrics and Adolescent Medicine, LKH Hochsteiermark, 8700 Leoben, Austria

In late 2019, heavy restrictions were enforced on public activities worldwide to diminish the spread of the severe acute respiratory syndrome coronavirus 2 (SARS-CoV-2) virus [1].

These restrictions, which included school closures and strictly limited sports and leisure opportunities, particularly impacted children and adolescents [2]. Lifestyle changes in children and adolescents led to reduced physical activity levels [2, 3], increased sedentary behavior [3, 4], changes in eating habits, and changes in sleep habits [3]. These subsequently affected important health parameters, such as body mass index (BMI) and cardiorespiratory fitness (CRF) [5–8]. We previously reported a reduction in CRF and an increase in BMI in 7–9-year-old children in Austria after the implementation of COVID-19-related restrictions in schools [9–11].

Here, we report how these important health markers [12, 13] developed in this cohort of children after the relaxation of COVID-19 mitigation measures. We analyzed data from September 2019 (pre-COVID-19), June 2021 (shortly after the abolishment of COVID-19-related restrictions), and June 2022 (1 year after the termination of mitigation measures). The study was registered in the German Clinical Trials Registry and approved by the local Research Ethics Committee (GZ. 39/23/63 ex 2018/19). Informed consent was obtained from the parents or legal guardians of the participants. For monitoring CRF, we used the 6-min run test, and BMI was standardized using Austrian reference centile curves, as described by the novel Austrian fitness monitoring tools (AUT FIT) [14]. Changes in the period during the stringent mitigation measures (Sept 2019–June 2021, P1), for the period after the relaxation of the stringent measures (June 2021–June 2022, P2), and for the whole study period (Sept 2019–June 2022, WSP) were calculated. We examined differences in subgroups by gender and sports club membership. Statistical analysis was performed using SPSS software (Version 28). All the tests were two-sided; a *P* value < 0.05 was considered significant.

A total of 331 children (mean age in September 2019 7.7 ± 0.4 years, range = 7–9 years) participated at all measurement time points. Among them, 159 (48.0%) were girls, and 146 (44.1%) were members of a sports club. Between September 2019 and June 2022 (WSP), the BMI score increased from 22.19 to 22.56 (*P* < 0.001). The highest BMI was observed at the end of P1 in June 2021 (22.82), while after the relaxation of the restrictions, BMI showed a significant decrease (*P* = 0.001) (Fig. 1, Table 1).

**Fig.1:**
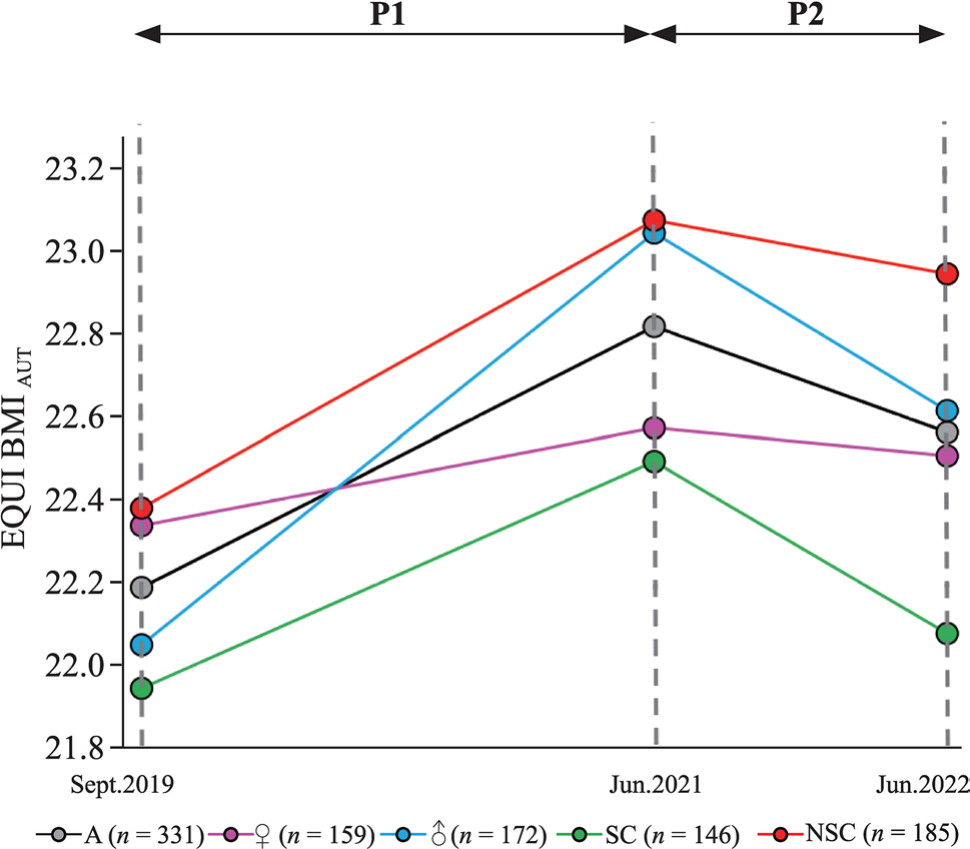
Changes in body mass index (EQUI BMI_AUT_) from September 2019 to June 2022. *P1* period with stringent mitigation measurement, *P2* period after relaxation of mitigation measurements, *BMI* body mass index, *EQUI BMI*_*AUT*_ equivalent BMI based on Austrian reference centile curves passing through adult BMI values, *A* all, ♀ girls, ♂ boys, *SC* member sports club, *NSC* not member sports club

**Table 1:** Data for BMI and CRF over measurement time points Sep19, June 21 and June 22

Variables	Groups	Sep 2019	*P* value^a^		*P* value^a^		*P* value^a^
			P1	June 2021	P2	June 2022	WSP
EQUI BMI_AUT_, mean (SD)	All (*n* = 331)	22.19 (3.27)	** < 0.001**	22.82 (3.50)	**0.001**	22.56 (3.55)	** < 0.001**
	Girls (*n* = 159)	22.34 (3.67)	0.120	22.57 (3.82)	> 0.999	22.50 (3.90)	0.483
	Boys (*n* = 172)	22.05 (2.85)	** < 0.001**	23.04 (3.17)	** < 0.001**	22.61 (3.20)	** < 0.001**
	Sports club (*n* = 146)	21.94 (2.52)	** < 0.001**	22.49 (2.76)	**0.003**	22.08 (2.73)	0.714
	No sports club (*n* = 185)	22.38 (3.75)	** < 0.001**	23.07 (3.97)	0.207	22.94 (4.05)	** < 0.001**
6MR SDS, mean (SD)	All (*n* = 331)	0.46 (1.10)	** < 0.001**	− 0.30 (1.11)	** < 0.001**	− 0.07 (1.19)	** < 0.001**
	Girls (*n* = 159)	0.28 (1.05)	** < 0.001**	− 0.59 (1.06)	**0.002**	− 0.28 (1.17)	** < 0.001**
	Boys (*n* = 172)	0.63 (1.12)	** < 0.001**	− 0.04 (1.10)	0.085	0.13 (1.18)	** < 0.001**
	Sports club (*n* = 146)	0.78 (1.05)	** < 0.001**	0.07 (1.03)	0.065	0.25 (1.17)	** < 0.001**
	No sports club (*n* = 185)	0.21 (1.07)	** < 0.001**	− 0.59 (1.08)	**0.002**	− 0.32 (1.15)	** < 0.001**

Bold values indicate significant differences

*P1* period during the stringent mitigation measures (Sept 2019 to June 2021), *P2* period after relaxation of the stringent measures (June 2021–June 2022), *WSP* whole study period (Sept 2019 to June 2022), *BMI* body mass index, *CRF* cardiorespiratory fitness, *EQUI BMI*_*AUT*_ equivalent BMI based on Austrian reference centile curves passing through adult BMI values, *6MR* six-minute run based on the reference values of the Düsseldorfer Modell, *SDS* standard deviation scores, *SD* standard deviation

^a^Adjusted for multiple comparisons using Bonferroni correction

Significant gender differences were found. Changes in BMI among girls were not statistically significant in any period. In boys, there were highly significant increases in BMI values during P1 (*P* < 0.001), followed by a strong decrease in P2 (*P* < 0.001). However, in June 2022, BMI in boys was still higher than in September 2019 (*P* < 0.001).

Both children with and without sports club membership showed a highly significant (*P* < 0.001) increase in BMI values in P1. In P2, a pronounced reduction (*P* = 0.003) was found in children who were members of a sports club. In contrast, children without sports club membership showed only a small, nonsignificant (*P* = 0.21) reduction in BMI values during P2, with no return to baseline values by the end of the observation period (June 2022).

When analyzing CRF, we found an extreme decrease during P1 (*P* < 0.001) in all subgroups (Fig. 2, Table 1). After the cessation of stringent mitigation measures during P2, significant improvements were found (*P* < 0.001) in the total group. Improvement was statistically significant in the subgroup of girls (*P* = 0.002) and in children without sports club membership (*P* = 0.002); however, this improvement was nonsignificant in boys (*P* = 0.08) and in children attending a sports club (*P* = 0.08, Fig. 2, Table 1). None of the subgroups returned to pre-COVID levels of CRF. Further information is given in the supplementary material in Supplementary Tables 1–6.

**Fig. 2:**
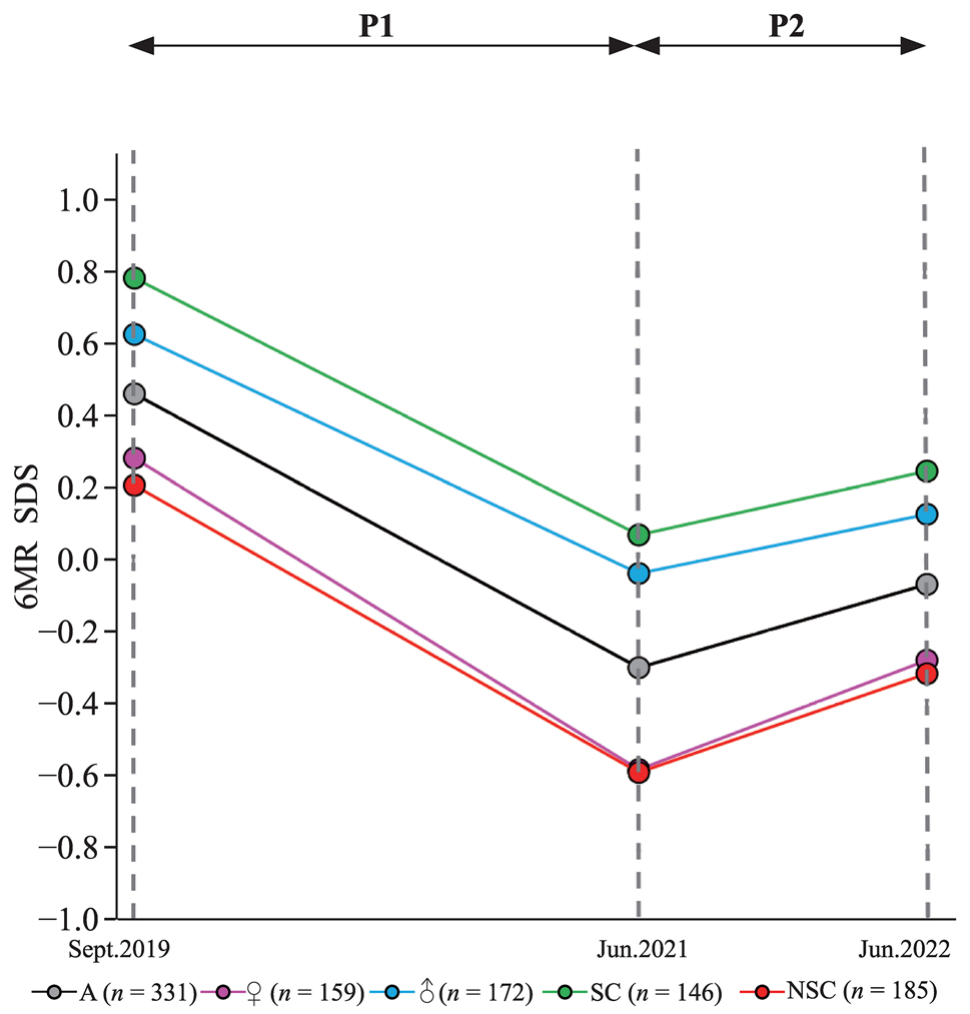
Changes in cardiorespiratory fitness (6MR) from September 2019 to June 2022. *P1* period with stringent mitigation measurement, *P2* period after relaxation of mitigation measurements, *6MR* six-minute run, *SDS* standard deviation score, *A* all, ♀ girls, ♂

Our observation of increasing BMI and decreasing CRF during P1 and improvement in both outcomes during P2 suggests that the negative effects of the pandemic and its mitigation measures on BMI are reversible, at least in primary school children.

However, it is important to note that a full reversal of the negative effects of the pandemic was not achieved, particularly in boys. This highlights the need for further action in addressing these issues in the future. Failure to do so might result in long-term deleterious public health consequences, most likely resulting in high socioeconomic burden and possibly even costing numerous life years.

Together with other studies [15, 16], our findings provide a basis for policymakers to more carefully consider the advantages and disadvantages of virus mitigation measures in the future. The benefit of such measures in terms of virus transmission must be balanced against long-term sequelae, such as BMI increase and fitness decrease as markers of physical health. The continued performance of physical activities should be considered as important as infection prevention, especially in school settings. For possible future pandemic periods, any associated closures of sports clubs and public playgrounds should at least guarantee the minimum level of physical activity recommended by the World Health Organization (60 minutes of moderate exercise daily) [17], preferably outdoors during school hours.

To continuously diminish the negative effects of the (hopefully) vanishing COVID-19 pandemic, a variety of school-based interventions should be scheduled. These measures should include a daily physical activity unit, healthy lunch, and the implementation of a health monitoring system in schools to verify the efficacy of such measures. Moreover, participation in sports clubs should be actively encouraged for school children and adolescents. Ultimately, policymakers need to recognize the importance of such programs, actively promoting and supporting their implementation.

### Supplementary Information

Below is the link to the electronic supplementary material.

Supplementary file1 (DOCX 52 KB)

## Data Availability

Data can be requested from the corresponding author and are provided free of charge for non-commercial purposes.
