# Supplementary material for: Changes in children’s cardiorespiratory fitness and body mass index over the course of the COVID-19 pandemic: a 34-month longitudinal study of 331 primary school children
Source: World J Pediatr. 2023 Nov 26;20(2):185–8. doi: 10.1007/s12519-023-00772-0 (PMC10884133; doi:10.1007/s12519-023-00772-0)
Supplement: Supplementary file 1 — Supplementary file1 (DOCX 52 KB) [file 12519_2023_772_MOESM1_ESM.docx]

**Supplements**

**Table S1.** Characteristics of the total sample study population vs. loss to follow-up.

**Table S2.** Number of study participants.

**Table S3.** Descriptive statistics of BMI, CRF and weight classification for all seven measurement time points.

**Table S4.** Mixed ANOVAS for BMI and CRF data over measurement time points T1, T2, and T3.

**Table S5.** Post hoc tests of EQUI BMI_AUT_ for the main effect of time and interactions for time*school group and time*sports club based on estimated marginal means.

**Table S6.** Post hoc tests of 6MR for the main effect of time and interactions for time*school group and time*sports club membership based on estimated marginal means.

This supplementary material has been provided by the authors to give readers additional information about their work.

**Table S1.** Characteristics of the total sample study population vs. loss to follow-up.

| **Variable** | **Study Population**  **N=331** | **Loss to Follow-up**  **N=86** | ***χ*^2^** | ***t*** | ***p*-lvl** | ***p*-Value** |
| --- | --- | --- | --- | --- | --- | --- |
| **Age (years)** | 7.7 (0.4) | 7.8 (0.5) |  | –1.828 |  | 0.07 |
| **Female gender** | 159 (48.0%) | 44 (53.5%) | 0.812 |  |  | 0.37 |
| **Sports club** | 146 (44.1%) | 33 (38.4%) | 0.917 |  |  | 0.34 |
| **EQUI BMI_AUT_, mean (SD)** | 22.18 (3.27) | 22.17 (3.93) |  | 0.046 |  | 0.96 |
| **6MR SDS (DüMo), mean (SD)** | 0.46 (1.10) | 0.12 (1.07) |  | 2.578 | * | 0.010 |
| Data are the *n* (%) or mean (SD). Sample size for study population, *n* = 331; for loss to follow-up, *n* = 86. *χ*^2^ = chi-square test value; *t* = *t*-test; *p*-lvl (*p-*value level), * *p* < 0.05; BMI = body mass index., EQUI BMI_AUT_ = equivalent BMI based on Austrian reference centile curves passing through adult BMI values; 6MR = six-minute run; SD = standard deviation; SDS = standard deviation score; DüMo = Düsseldorfer Modell. | | | | | | |

**Table S2.** Number of study participants.

| **Variable** | **All** |  | **Girls** | **Boys** | **Sports Club** | **No Sports Club** |
| --- | --- | --- | --- | --- | --- | --- |
| BMI | 331 |  | 159 | 172 | 146 | 185 |
| 6MR |  |  |  |  |  |  |

BMI = body mass index; 6MR = six-minute run.

**Table S3.** Descriptive statistics of BMI, CRF and weight classification for all seven measurement time points.

| **Variable** | **Group** | **Sep.19** | **Jun.20** | **Sep.20** | **Mär.21** | **Jun.21** | **Sep.21** | **Jun.22** |
| --- | --- | --- | --- | --- | --- | --- | --- | --- |
| **EQUI BMI_AUT_, mean (SD)** | All (n=331) | 22.19 (3.27) | 22.44 (3.51) | 22.63 (3.49) | 22.77 (3.58) | 22.82 (3.50) | 22.58 (3.47) | 22.56 (3.55) |
|  | Girls (n=159) | 22.34 (3.67) | 22.48 (3.82) | 22.56 (3.92) | 22.51 (3.99) | 22.57 (3.82) | 22.46 (3.80) | 22.50 (3.90) |
|  | Boys (n=172) | 22.05 (2.85) | 22.40 (3.20) | 22.70 (3.06) | 23.00 (3.14) | 23.04 (3.17) | 22.69 (3.13) | 22.61 (3.20) |
|  | Sports Club (n=146) | 21.94 (2.52) | 22.01 (2.93) | 22.30 (2.78) | 22.50 (2.90) | 22.49 (2.76) | 22.12 (2.77) | 22.08 (2.73) |
|  | No Sports Club (n=185) | 22.38 (3.75) | 22.78 (3.88) | 22.89 (3.96) | 22.97 (4.03) | 23.07 (3.97) | 22.94 (3.90) | 22.94 (4.05) |
| **6MR, mean (SD) in m** | All (n=331) | 903 (135) | n.t. | 804 (126) | 824 (145) | 851 (153) | 850 (169) | 935 (171) |
|  | Girls (n=159) | 845 (112) | n.t. | 759 (106) | 766 (125) | 782 (127) | 782 (139) | 865 (147) |
|  | Boys (n=172) | 957 (132) | n.t. | 845 (130) | 876 (142) | 915 (148) | 913 (170) | 1000 (166) |
|  | Sports Club (n=146) | 954 (128) | n.t. | 842 (133) | 873 (146) | 913 (144) | 907 (176) | 993 (169) |
|  | No Sports Club (n=185) | 863 (126) | n.t. | 774 (113) | 785 (132) | 803 (143) | 805 (148) | 890 (158) |
| **6MR SDS (DüMo), mean (SD)** | All (n=331) | 0.46 (1.10) | n.t. | -0.51 (0.92) | -0.47 (1.04) | -0.30 (1.11) | -0.46 (1.19) | -0.07 (1.19) |
|  | Girls (n=159) | 0.28 (1.05) | n.t. | -0.63 (0.88) | -0.67 (1.02) | -0.59 (1.06) | -0.72 (1.11) | -0.28 (1.17) |
|  | Boys (n=172) | 0.63 (1.12) | n.t. | -0.40 (0.95) | -0.28 (1.04) | -0.04 (1.10) | -0.21 (1.22) | 0.13 (1.18) |
|  | Sports Club (n=146) | 0.78 (1.05) | n.t. | -0.29 (0.96) | -0.18 (1.04) | 0.07 (1.03) | -0.12 (1.23) | 0.25 (1.17) |
|  | No Sports Club (n=185) | 0.21 (1.07) | n.t. | -0.68 (0.86) | -0.69 (0.99) | -0.59 (1.08) | -0.72 (1.09) | -0.32 (1.15) |

Data are the No. (%) or mean (SD). SY21–22 = school year 2021–2022; EQUI BMI_AUT_ = equivalent BMI based on Austrian reference centile curves passing through adult BMI valuesSD = standard deviation; 6MR = six-minute run; SDS = standard deviation score; DüMo = Düsseldorfer Modell; n. t.= no testing;

**Table S4.** Mixed ANOVAS for BMI and CRF data over measurement time points T1, T2, and T3.

|  |  | **Effects** | **df** | **F** | ***P* Value** | **η_p_^2^** | **Power^a^** |
| --- | --- | --- | --- | --- | --- | --- | --- |
| **EQUI BMI_AUT_** | Between-subjects effects | Sports Club | 1 | 3.893 | 0.049 | 0.012 | 0.503 |
|  |  | Gender | 1 | 0.912 | 0.340 | 0.003 | 0.159 |
|  |  | Sports Club*Gender | 1 | 1.005 | 0.317 | 0.003 | 0.170 |
|  |  | Error | 327 |  |  |  |  |
|  | Within-subjects effects | Time (T1-T2-T3) | 1.553 | 34.520 | <.001 | 0.095 | 1.000 |
|  |  | Time*Sports Club | 1.553 | 5.499 | 0.008 | 0.017 | 0.779 |
|  |  | Time*Gender | 1.553 | 12.970 | <.001 | 0.038 | 0.989 |
|  |  | Time*Sports Club*Gender | 1.553 | 9.323 | <.001 | 0.028 | 0.949 |
|  |  | Error (Time) | 507.966 |  |  |  |  |
| **6MR SDS** | Between-subjects effects | Sports Club | 1 | 22.167 | <.001 | 0.063 | 0.997 |
|  |  | Gender | 1 | 8.876 | 0.003 | 0.026 | 0.844 |
|  |  | Sports Club*Gender | 1 | 3.243 | 0.073 | 0.010 | 0.435 |
|  |  | Error | 327 |  |  |  |  |
|  | Within-subjects effects | Time (T1-T2-T3) | 2 | 85.949 | <.001 | 0.208 | 1.000 |
|  |  | Time*Sports Club | 2 | 0.119 | 0.888 | <.001 | 0.068 |
|  |  | Time*Gender | 2 | 1.535 | 0.216 | 0.005 | 0.327 |
|  |  | Time*Sports Club*Gender | 2 | 0.464 | 0.629 | 0.001 | 0.126 |
|  |  | Error (Time) | 654 |  |  |  |  |

^a^ Observed power computed using alpha = 0.05. ANOVA = analysis of variance; BMI = body mass index; df = degrees of freedom; *F* = test statistic; $\boldsymbol{\eta}_{\boldsymbol{p}}^{\boldsymbol{2}}$ = partial eta square; EQUI BMI_AUT_ = equivalent BMI based on Austrian reference centile curves passing through adult BMI values; 6MR = six-minute run;; T1 = baseline measurements in September 2019; T2 = follow-up measurements in June 2021 after the stringent mitigation measurements; T3 = follow-up measurements in June 2022 after a period of relaxation of the COVID-19 related mitigation measurements.

**Table S5.** Post hoc tests of EQUI BMI_AUT_ for the main effect of time and interactions for time*school group and time*sports club based on estimated marginal means.

|  | | **Pairwise comparisons** | **Mean diff (95% CI)** | | | | | | **SE** | ***P* Value^a^** | **p-lvl** |
| --- | --- | --- | --- | --- | --- | --- | --- | --- | --- | --- | --- |
| **EQUI BMI_AUT_** | Time | T1 to T2 | -,652^*^ | ( | -0.843 | to | -0.460 | ) | 0.080 | <.001 | *** |
|  |  | T1 to T3 | -,433^*^ | ( | -0.666 | to | -0.201 | ) | 0.097 | <.001 | *** |
|  |  | T2 to T3 | ,218^*^ | ( | 0.078 | to | 0.359 | ) | 0.058 | 0.001 | ** |
|  | Time*Girls | T1 to T2 | -0.246 | ( | -0.534 | to | 0.041 | ) | 0.119 | 0.12 |  |
|  |  | T1 to T3 | -0.203 | ( | -0.552 | to | 0.145 | ) | 0.145 | 0.48 |  |
|  |  | T2 to T3 | 0.043 | ( | -0.168 | to | 0.254 | ) | 0.088 | >.99 |  |
|  | Time*Boys | T1 to T2 | -1,057^*^ | ( | -1.311 | to | -0.803 | ) | 0.106 | <.001 | *** |
|  |  | T1 to T3 | -,663^*^ | ( | -0.971 | to | -0.355 | ) | 0.128 | <.001 | *** |
|  |  | T2 to T3 | ,394^*^ | ( | 0.208 | to | 0.581 | ) | 0.077 | <.001 | *** |
|  | Time*Sports Club | T1 to T2 | -,473^*^ | ( | -0.766 | to | -0.181 | ) | 0.122 | <.001 | *** |
|  |  | T1 to T3 | -0.174 | ( | -0.529 | to | 0.181 | ) | 0.148 | 0.71 |  |
|  |  | T2 to T3 | ,299^*^ | ( | 0.084 | to | 0.514 | ) | 0.089 | 0.003 | ** |
|  | Time*No Sports Club | T1 to T2 | -,830^*^ | ( | -1.077 | to | -0.582 | ) | 0.103 | <.001 | *** |
|  |  | T1 to T3 | -,692^*^ | ( | -0.992 | to | -0.391 | ) | 0.125 | <.001 | *** |
|  |  | T2 to T3 | 0.138 | ( | -0.044 | to | 0.320 | ) | 0.076 | 0.21 |  |

^a^ Adjusted for multiple comparisons using Bonferroni correction. *p*-lvl (*p*-value level) = ** *p* < 0.01, and *** *p* < 0.001. BMI = body mass index; CI = confidence interval; EQUI BMI_AUT_ = equivalent BMI based on Austrian reference centile curves passing through adult BMI values; mean difference = mean difference based on the estimated marginal means; *p*-lvl = significance level; SE = standard error; T1 = baseline measurements in September 2019; T2 = follow-up measurements in June 2021 after the stringent mitigation measurements; T3 = follow-up measurements in June 2022 after a period of relaxation of the COVID-19 related mitigation measurements.

**Table S6.** Post hoc tests of 6MR for the main effect of time and interactions for time*school group and time*sports club membership based on estimated marginal means.

|  | | **Pairwise comparisons** | **Mean diff (95% CI)** | | | | | | **SE** | ***P* Value^a^** | **p-lvl** |
| --- | --- | --- | --- | --- | --- | --- | --- | --- | --- | --- | --- |
| **6MR SDS (DüMo)** | Time | T1 to T2 | ,783^*^ | ( | 0.635 | to | 0.932 | ) | 0.062 | <.001 | *** |
|  |  | T1 to T3 | ,546^*^ | ( | 0.395 | to | 0.697 | ) | 0.063 | <.001 | *** |
|  |  | T2 to T3 | -,237^*^ | ( | -0.380 | to | -0.095 | ) | 0.059 | <.001 | *** |
|  | Time*Girls | T1 to T2 | ,890^*^ | ( | 0.668 | to | 1.112 | ) | 0.092 | <.001 | *** |
|  |  | T1 to T3 | ,588^*^ | ( | 0.362 | to | 0.815 | ) | 0.094 | <.001 | *** |
|  |  | T2 to T3 | -,301^*^ | ( | -0.515 | to | -0.088 | ) | 0.089 | 0.002 | ** |
|  | Time*Boys | T1 to T2 | ,677^*^ | ( | 0.480 | to | 0.873 | ) | 0.082 | <.001 | *** |
|  |  | T1 to T3 | ,504^*^ | ( | 0.303 | to | 0.704 | ) | 0.083 | <.001 | *** |
|  |  | T2 to T3 | -0.173 | ( | -0.362 | to | 0.016 | ) | 0.078 | 0.08 |  |
|  | Time*Sports Club | T1 to T2 | ,777^*^ | ( | 0.550 | to | 1.003 | ) | 0.094 | <.001 | *** |
|  |  | T1 to T3 | ,568^*^ | ( | 0.337 | to | 0.799 | ) | 0.096 | <.001 | *** |
|  |  | T2 to T3 | -0.209 | ( | -0.426 | to | 0.009 | ) | 0.090 | 0.07 |  |
|  | Time*No Sports Club | T1 to T2 | ,790^*^ | ( | 0.598 | to | 0.981 | ) | 0.080 | <.001 | *** |
|  |  | T1 to T3 | ,524^*^ | ( | 0.329 | to | 0.720 | ) | 0.081 | <.001 | *** |
|  |  | T2 to T3 | -,266^*^ | ( | -0.450 | to | -0.081 | ) | 0.077 | 0.002 | ** |

^a^ Adjusted for multiple comparisons using Bonferroni correction. *p*-lvl (*p*-value level) = ** *p* < 0.01, and *** *p* < 0.001; CI = confidence interval; 6MR = six-minute run; DüMo = Düsseldorfer Modell; Mean Difference = mean difference based on the estimated marginal means; *p*-lvl = significance level; SE = standard error; T1 = baseline measurements in September 2019; T2 = follow-up measurements in June 2021 after the stringent mitigation measurements; T3 = follow-up measurements in June 2022 after a period of relaxation of the COVID-19 related mitigation measurements.
